# 966. Improving Occupation-based Care at a Southern Regional Burn Center: Results of a Performance Improvement Initiative

**DOI:** 10.1093/jbcr/irag033.563

**Published:** 2026-04-06

**Authors:** Jill M Cancio, Alexis L Mize

**Affiliations:** US Army Institute of Surgical Research Burn Center, Joint Base San Antonio, Fort Sam Houston, TX; Baylor University, San Antonio, TX

## Abstract

**Introduction:**

Burn injuries cause complex physical, psychological, and occupational disruption, requiring comprehensive rehabilitation. Occupation-based interventions (OBIs)—those grounded in purposeful, real-life activity—have been shown to enhance functional outcomes and promote psychological recovery. In high-acuity environments like our Burn Center, occupational therapy (OT) practice often defaults to biomechanical or preparatory approaches due to barriers such as medical instability, documentation demands, and cultural norms. There is limited research exploring structured methods to integrate OBIs into acute burn rehabilitation. Purpose is to describe findings from a performance improvement (PI) project intended to understand occupation-based care and improve OBIs within our Burn Center’s OT department.

**Methods:**

FOCUS-PDCA PI model was used. Baseline data were collected on patients being treated by occupational therapists at a Burn Center in the southern region of the US from 18MAR2025 to 02APR2025 and compared to post-intervention data collected from 07JUL2025 to 18JUL2025. The following evaluation metrics were selected: observed OT sessions scored via the Occupation-Based Practice Assessment (OBPA), and rehabilitation staff surveys. The plan for improvement included staff in-services, training, and modification of electronic medical record templates, publication of a weekly newsletter, and development of occupation-based activity kits (OBK). Nonparametric tests were used as indicated.

**Results:**

Data were collected on 15 patients for a total of 132 treatment observations at baseline and 11 patients with a total of 126 treatment observations at the post-intervention timepoint. We found that (1) median OPBA total scores improved significantly in the post-intervention period (20 to 22, z = 3.429 [*p*<.001], effect size [ES] r = 0.2); (2) specifically, improvements in the burn intensive care unit median OPBA scores were statistically significant (17 to 21, z = 2.713 [*p*=.007], ES r = 0.3); (3) significantly more therapists could articulate the meaning of occupation-based care at the post-intervention timepoint (18% vs 82% [*p*<.05]); and (4) 100% of therapists surveyed post-intervention noted clients responded “very positively” and demonstrated improved motivation, task persistence, and self-expression with use of the OBKs vs. usual treatment modalities.

**Conclusions:**

The project successfully enhanced staff understanding and use of OBIs at our Burn Center.

**Applicability of Research to Practice:**

This fostered a cultural shift in clinical reasoning, making occupation-based care more accessible, visible, and sustainable. Findings suggest that when equipped with the right systems and supports, OBI therapy at our Burn Center is possible even in the most medically complex environments.

**Funding for the study:**

N/A.

